# Evaluation of multiplex ligation dependent probe amplification (MLPA) for identification of acute lymphoblastic leukemia with an intrachromosomal amplification of chromosome 21 (iAMP21) in a Brazilian population

**DOI:** 10.1186/s13039-015-0147-2

**Published:** 2015-06-10

**Authors:** Gerhard Fuka, Tállita M. Farias-Vieira, Leticia Hummel, Caroline B. Blunck, Júlio C. Santoro, Eugênia Terra-Granado, Thayana Conceição Barbosa, Mariana Emerenciano, Maria S. Pombo-de-Oliveira

**Affiliations:** Pediatric Hematology-Oncology Program, Research Center, Instituto Nacional de Câncer (INCA), Rua André Cavalcanti, 37, Rio de Janeiro, RJ 20231-050 Brazil

**Keywords:** B-cell precursor acute lymphoblastic leukemia (BCP-ALL), Intrachromosomal amplification of chromosome 21 (iAMP21), Multiplex ligation dependent probe amplification (MLPA), Fluorescence *in situ* hybridization (FISH)

## Abstract

**Background:**

An intrachromosomal amplification of chromosome 21 (iAMP21) defines a unique subgroup of B-cell precursor acute lymphoblastic leukemia (BCP-ALL). The finding of three or more extra copies of the *RUNX1* gene by fluorescence *in situ* hybridization (FISH) is internationally used to define an iAMP21. Genomic profiling of chromosome 21 has been suggested for assisting diagnostic case identification. Due to limitations of comparative genomic hybridization, in terms of a routine application as first line-screening tests we evaluated the multiplex ligation-dependent probe amplification (MLPA) SALSA P327_A1 and P327_B1 probe sets for detecting chromosome 21 copy number alterations in Brazilian childhood BCP-ALL.

**Results:**

In 74 out of 368 patients gain of genetic material was detected. For data confirmation *RUNX1* directed FISH was performed. Cells with ≥5 *RUNX1* signals (n = 9) were considered as “true iAMP21” while <5 *RUNX1* signals (n = 41) were counted as evidence for additional copies of intact chromosomes 21. All patients with an iAMP21 had high MLPA peak ratios (≥1.8), while the majority of patients with <5 *RUNX1* presented low MLPA peak ratios (<1.8). Observed differences gained statistical strength by comparing probes located within the common region of amplification. Next, a principal component analysis was performed in order to illustrate distribution of cases according to their MLPA peak profile in two dimensions. Cases with an iAMP21 mostly clustered together, however additional cases with <5 RUNX1 signals or no available FISH data located in proximity.

**Conclusions:**

MLPA qualified as a high throughput technique that could be employed in future studies for a critical comparison with data obtained by FISH, especially in cases where metaphase nuclei are not available. Taking submicroscopic aberrations into account examined by MLPA, cases exhibiting an “iAMP21 like” peak ratio profile but <5 *RUNX1* signals should be considered as candidates for this chromosomal abnormality.

**Electronic supplementary material:**

The online version of this article (doi:10.1186/s13039-015-0147-2) contains supplementary material, which is available to authorized users.

## Background

Cytogenetic and molecular studies have displayed among B-cell precursor-acute lymphoblastic leukemia (BCP-ALL) a distinct subtype, characterized by an intrachromosomal amplification of chromosome 21 (iAMP21) [1, 2].

Patients with an iAMP21 treated under standard risk regimens had an elevated relapse rate and a dismal prognosis compared to other BCP-ALL, hence risk directed treatment intensification has been recommended [3–7]. Amplification of 21q has also been found in other hematopoietic malignancies such as acute myeloid leukemia and disorders with complex karyotypes involving chromosome 21, supporting the hypothesis that gains within chromosome 21 interplay with the function of a specific set of genes [8]. Array based genomic analyses revealed that alterations on chromosome 21 are highly complex and suggested to arise from breakage-fusion bridge cycles followed by chromothripsis and other complex structural rearrangements on chromosome 21 [9–11]. Multiple regions of gains, amplifications, inversions and deletions with a high level of individuality between different patients have been described. Patients exhibited a common region of amplification (CRA, covering a region of 6 MB) as well as in most cases a common region of deletion (CRD, covering a region of 9 MB) [10]. The CRA, located between 32.8-37.9 MB on chromosome 21, consistently showed the most amplified as well as over-expressed regions, encoding for genes that play important roles in leukemia such as *RUNX1*, *DYRK1A* and *ETS2* [11].

The method nowadays internationally recommended to identify an iAMP21 is fluorescence *in situ* hybridization (FISH) using probes directed to the *Runt Related Transcription Factor 1 (RUNX1*) gene, present by three or more extra copies on a single abnormal chromosome 21 [10, 12]. However, case interpretation needs to be done cautiously, particularly whenever FISH was performed from interphase nuclei because additional copies of the *RUNX1* genes are also present in high hyperdiploid karyotypes. In light of such considerations, genomic analyses of chromosome 21 might be taken into account for confirming the accuracy of iAMP21 diagnosis [13]. Multiplex ligation-dependent probe amplification (MLPA) assays represent a quick and cost effective alternative to array based techniques allowing screening of large cohorts for sub microscopic copy number changes. By quantification of multiple gene sequences on multiple sites on a previously defined chromosomal region such as an abnormal chromosome 21 it permits data comparison with other molecular techniques [14].

In this work, we evaluated MLPA for detecting an iAMP21 in a cohort of Brazilian childhood BCP-ALL and compared data with those obtained from FISH, conventionally detecting *RUNX1* copy numbers. To our knowledge, this is the first published report where the SALSA MLPA P327_A1 and P327_B1 probe sets were tested in order to identify copy number alterations (CNAs) in a subset of genes, demonstrating its value as a tool for screening large cohorts of patients.

## Results

Demographic and clinical characteristics of 368 BCP-ALL alleged according to designated criteria with the MLPA results are shown in Table 1. Most variables were equally distributed in negative and positive cases, but age between 2-10 years, common-ALL and standard prognostic risk was more prone in MLPA positive patients (p < 0.05). Blast cell counts were similar in both groups with an average of 82 % (range 67 % to 90 %) in the whole cohort.

**Table 1:** Baseline characteristics of BCP-ALL with and without chromosome 21 CNAs

Characteristics	MLPA negative	MLPA positive	*P*-value
	n (%)	n (%)	
Total	294 (80.0)	74 (20.0)	
Gender			
Male	169 (57.0)	42 (56.8)	
Female	125 (43.0)	32 (43.2)	0.999
Age range, (years)			
≤1	15 (5.1)	0 (0.0)	
2-10	185 (62.6)	62 (83.8)	
>10	94 (32.3)	12 (16.2)	0.001**
Skin color			
White	117 (42.5)	36 (50.0)	
Non-white	159 (57.5)	36 (50.0)	0.300
WBC, x10^9^/l			
Median	61.1	44.8	
Range	0.6-978.0	2.2-95.0	
≤20	153 (52.8)	40 (54.8)	
20-50	56 (19.3)	15 (20.5)	
>50	81 (27.9)	18 (24.7)	0.816
Immunophenotype			
Pro-B ALL	31 (10.5)	1 (1.4)	
Common-ALL	205 (69.8)	58 (78.3)	
Pre-B ALL	58 (19.7)	15 (20.3)	0.038*
NCI risk group			
Standard risk	141 (48.3)	46 (63.0)	
High risk	151 (51.7)	27 (37.0)	0.018*
Status			
Alive	28 (65.1)	21 (75.0)	
Dead	15 (34.9)	7 (25.0)	0.379

*n* number, *WBC* white blood cell count x10^9^/l

*p < 0.05; **p ≤ 0.01

**Fig. 1:**
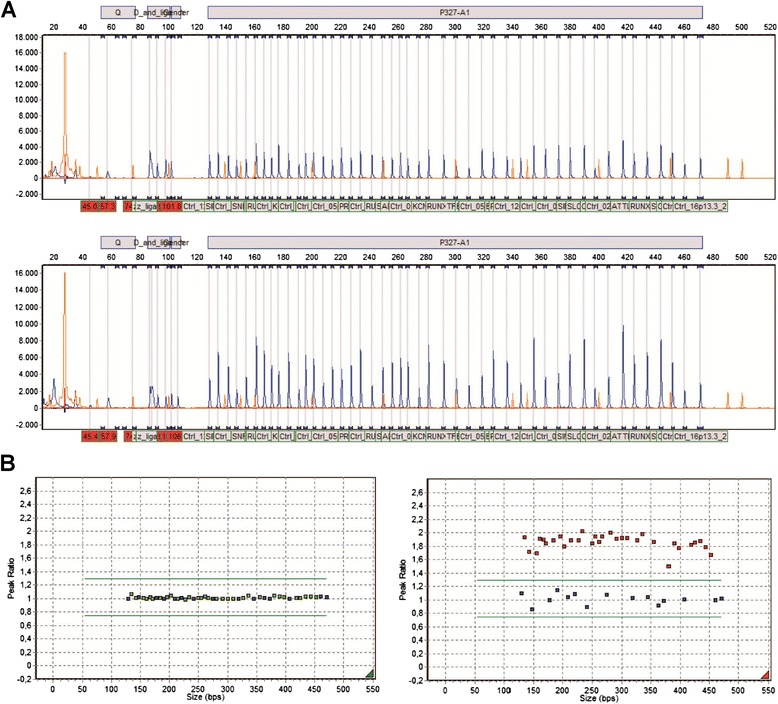
Representative MLPA data shown as screen shots from the Gene-Marker 2.2.0 analysis software. **a**. MLPA electropherograms of cord blood (CB) DNA (upper panel) compared to DNA from P4 (lower panel). Differences in peaks heights suggest altered dosages of corresponding exons depicted below. **b**. MLPA scatter plots for CB DNA (left panel) compared to P4 DNA (right panel). Reference probes (blue points) and test probes (green points) are within the normal range (located between green lines) for the CB sample, while test probes (red points) appear elevated for P4

Patient material was first subjected to MLPA using the SALSA MLPA P327_A1 probe set. Relative peak heights between 0.75 and 1.3 were considered normal, while those below 0.75 and above 1.3 indicated losses or gains of genomic material, respectively (Fig. 1). Two hundred and ninety-four patients had no copy number changes of chromosome 21 regions since test fragments binding on chromosome 21 and control fragments binding on other chromosomes appeared normal, whereas in 74 patients gain of genetic material on chromosome 21 was found. None of our patients harbored gene deletions on chromosome 21.

**Fig. 2:**
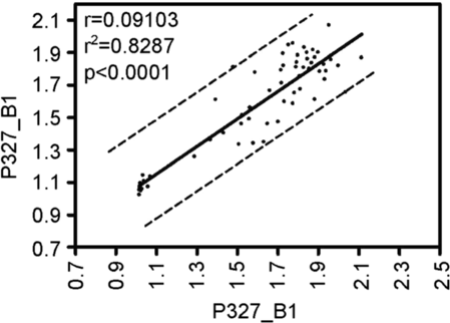
Pearson’s correlation coefficient analysis of data obtained from SALSA MLPA P327_A1 and P327_B1 probe sets. MLPA mean peak ratios over all chromosome 21 probes (black circles) were plotted for each patient; the black line depicts the linear correlation of data. Pearson correlation coefficients (r), r-squared (r^2^)

For data validation and sub-division of MLPA positive patients we next applied the SALSA MLPA P327_B1 probe set. Down syndrome (DS) patients and cytogenetically confirmed hyperdiploid ALL with extra copies of chromosomes 21 (Hyp + 21) were included as additional controls. We could not re-evaluate all 74 cases with chromosome 21 CNAs due to restrictions in the availability of DNA. However, for those patients subjected to analysis, no qualitative differences have been observed (n = 64); the presence or absence of CNAs on chromosome 21 has been confirmed. Pearson’s correlation coefficients (r = 0.09101, r^2^ = 0.8287, p < 0.0001), calculated to measure the linear dependence of variables and Kappa tests (κ = 0.661, SE = 0.083, 95 % CI = 0.498-0.825) to evaluate the number of observed agreements revealed only minor differences between average peak heights obtained from P327_A1 and P327_B1 probe sets (Fig. 2).

**Fig. 3:**
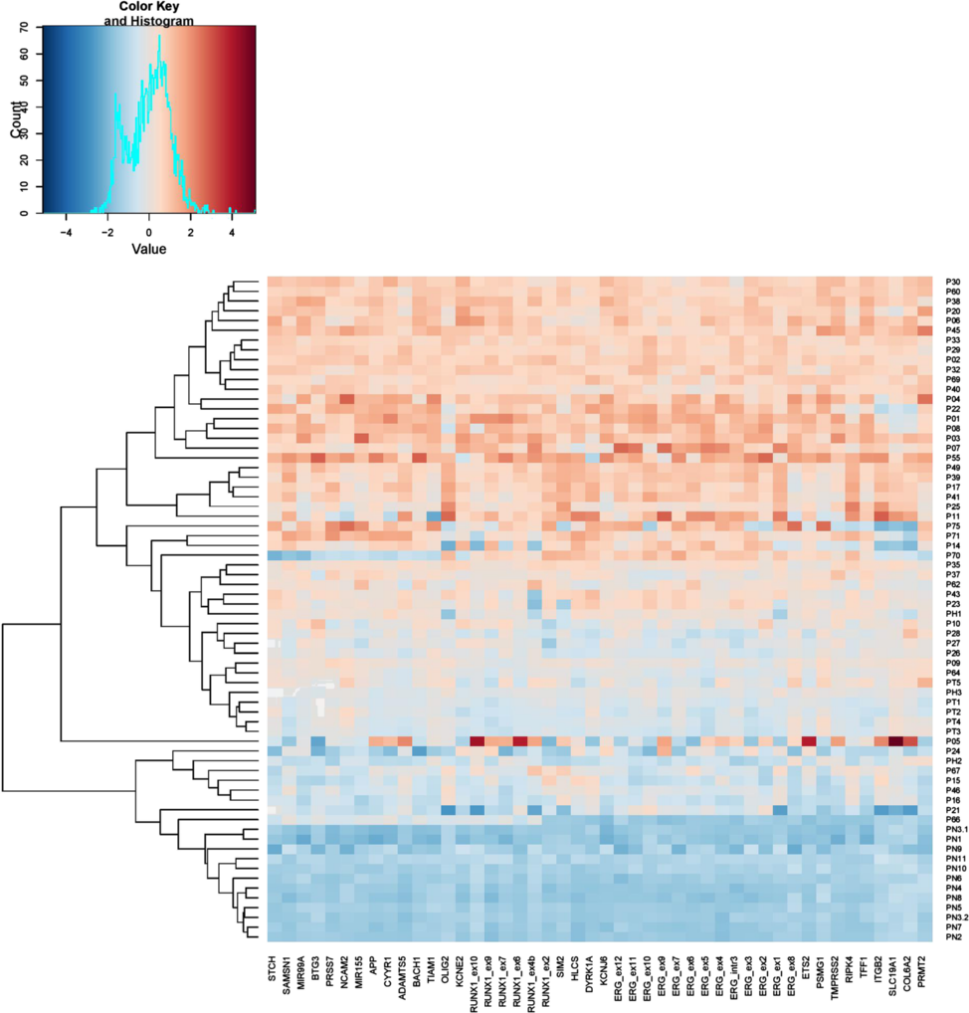
Hierarchical clustering of patients with and w/o chromosome 21 CNAs, DS and Hyp + 21 controls (n = 68). Data is arranged horizontally from centromeric to telomeric positions on chromosome 21 and vertically according to similarities in the MLPA peak ratio pattern (hierarchical clustering)

In order to understand gene CNAs distributions, MLPA results were illustrated in a heat map and arranged from centromeric to telomeric positions. MLPA peak heights allocated patients with chromosome 21 abnormalities into 3 main groups by a hierarchical clustering analysis (Fig. 3). Group 1 (bottom, n = 8) consisted of patients with slightly elevated MLPA peak ratios clustering in proximity to BCP-ALL without chromosome 21 CNAs; group 2 (middle, n = 15) of patients with intermediate peak ratios - similar to those observed in DS and Hyp + 21 - and, group 3 (top, n = 26) of patients with high or very high MLPA peak ratio profiles. Patient (P) 5 did not allocate into any of the three groups since its MLPA peak pattern demonstrated a higher variability than others.

**Fig. 4:**
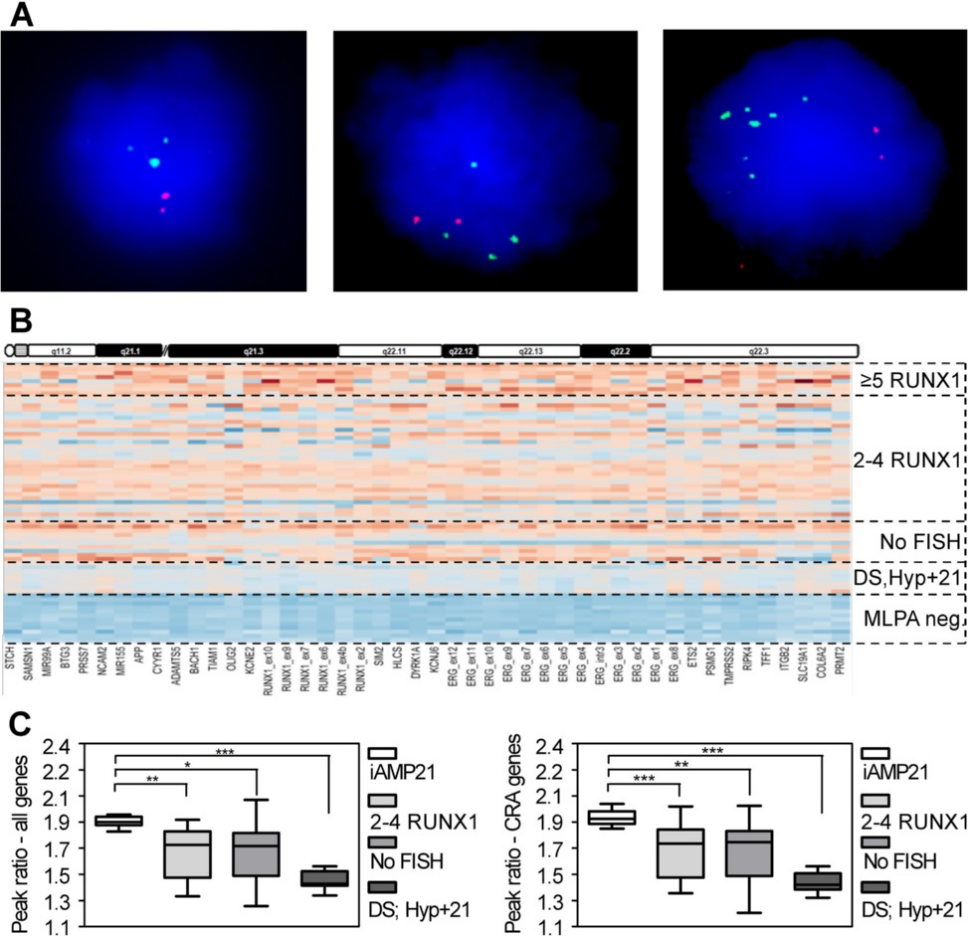
Subdivison of patients with CNAs of chromosome 21 by *RUNX1* directed FISH. **a**. Interphase FISH detecting ETV6 (red) and RUNX1 (green) alleles from patients with elevated MLPA peak ratios. The number of RUNX1 signals was counted as evidence to differentiate between BCP-ALL with additional copies of chromosome 21 (left; middle; 3–4 signals) and an iAMP21 (right; ≥5 signals). **b**. Heat map plotting MLPA peak ratios obtained from the P327_B1 probe set. Depicted are patients with 2–4 RUNX1 signals (n = 31), ≥5 RUNX1 signals (n = 8), no available FISH data (n = 10), DS and Hyp + 21 controls (n = 8) as well as normal MLPA peak ratios (n = 12). The scale from Fig. 3 was applied. **c**. Scatter plot diagrams showing mean peak ratios of patients in annotated groups over all chromosome 21 probes (left) and CRA probes (right). Dashed lines illustrate mean values of each patient group. *, P < 0.05; ***, **, P < 0.01; ***, P < 0.001 (unpaired *t*-test, two tailed)

According to availability of material FISH directed to the *RUNX1* gene was performed in 50 patients. Six patients had a normal FISH pattern with 2 signals of *RUNX1;* in 35 patients 3–4 signals for *RUNX1 -* while in 9 patients ≥5 signals for *RUNX1* were observed (Fig. 4a). Cells with 2–4 *RUNX1* signals most likely acquired one or two additional copies of chromosome 21 (even though not detectable in all cases by interphase FISH), while those with ≥5 *RUNX1* signals fulfilled the internationally adopted criteria for an iAMP21.

Patients with an iAMP21 had a mean age of 55 months (range 22–120) and a median WBC (white blood cell count) of 15.9 x 10^9^/l (range 7.2-92.0). According to National Cancer Institute (NCI) risk classification criteria, P5, P6 and P9 (33,3 %) allocated to the high risk group. P1, P3, P4 and P5 remained in complete remission until the last follow up in November 2014, while P8 and P9 succumbed to disease. For P2, P6, and P7 no follow up data was available.

**Fig. 5:**
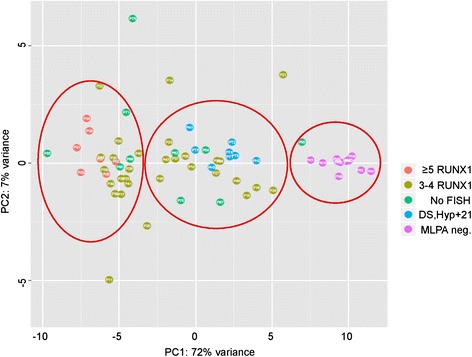
Principal component analysis of grouped patients. Individuals are distributed according to similarities in their MLPA peak ratio profiles and depicted by color-coded circles that designate certain patient groups. Red Circles demonstrate the presence of three main clusters

For data comparison of the methodologies MLPA and FISH patients were clustered according to the number of *RUNX1* signals (Fig. 4b). Mean MLPA peak ratios and standard deviations for chromosome 21 probes are provided in Additional file 1. MLPA peak ratios and in particular those of CRA genes separated cases with ≥5 *RUNX1* signals from cases with 2–4 *RUNX1* signals. In the latter group these were lower and in the same magnitude as controls such as patients with DS (PT1-5) and Hyp + 21 (PH1-3) (Fig. 4c). We next performed a principal component analysis in order to illustrate the distribution of cases according to their MLPA peak profile. Patients were grouped into (1) FISH confirmed iAMP21, (2) <5 *RUNX1* signals, (3) no FISH data, (4) DS and Hyp + 21, and (5) no chromosome 21 aberrations (Fig. 5). Sixty-three out of sixty-eight patients segregated into three clusters: The first one consisted of all patients from group 1 and two patients from group 3 and 4; the second of all patients from group 4 and most of the patients of group 2; and the third one of all patients from group 1 except P5. Interestingly, the latter cluster also contained some patients of group 2 and group 3 that located in close proximity to patients with an iAMP21; in other words exhibited an “iAMP21 like” MLPA peak profile.

Since we observed variations in MLPA peak heights between different patients, the correlation between *RUNX1, DYRK1A, ETS2* and *ERG* gene load and expression was tested. Delta threshold cycle (dCT) values for each patient are shown in Table 2. For illustration we stepwise arranged cases from the lowest to the highest MLPA mean peak ratios and depicted corresponding dCT values (Fig. 6). Positive correlation between the MLPA mean peak ratios and dCT values were observed, since those patients with high MLPA mean peak ratios were the ones with low gene expression. This observation particularly held true for the genes *RUNX1, DYRK1A,* and *ERG*.

**Table 2:** RT-qPCR data of patients with an iAMP21 for selected genes located in the CRA

#	dCT DYRK1A	dCT ERG	dCT ETS2	dCT RUNX1
P1	8.715	1.790	0.555	5.950
P2	11.625	7.655	6.430	12.195
P3	3.995	1.775	1.760	3.515
P4	7.885	5.380	4.155	7.250
P5	9.905	7.230	6.200	12.595
P6	9.815	6.665	6.200	11.095
P7	8.420	6.765	3.895	9.630
P8	11.010	5.635	4.845	11.000

**Fig. 6:**
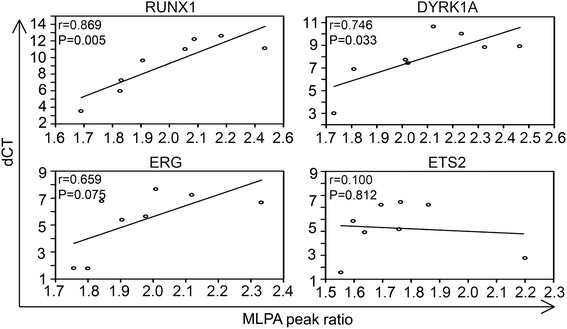
Correlation of MLPA mean peak ratios and gene expression in patients with an iAMP21. Scatter plots demonstrate the relation between mean MLPA peak ratios and mRNA expression of genes located within the CRA. Each circle represents data of one individual. Similarities between variables are depicted by linear regression (lines), Pearson correlation coefficients (r) and p-values

## Discussion

In this study, we evaluated MLPA as a tool to detect an iAMP21 in a cohort of BCP-ALL, providing first data of this leukemia entity in a Brazilian BCP-ALL cohort. The information about gene CNA distributions on chromosome 21 was re-tested. Nine out of 368 BCP-ALL patients (2.4 %) were identified with an iAMP21 according to criteria specified recently [13]*.* Taking submicroscopic aberrations into account examined by MLPA, additional cases presenting high MLPA peak ratios of genes within the CRA region or exhibiting an “iAMP21 like” peak ratio profile of chromosome 21 genes might be considered as positive for this chromosomal abnormality and subjected to further genomic tests.

In the past 10 years MLPA was successfully applied as a tool in cancer research, providing precise information on increased or decreased copy numbers at specific loci [14]. More recently it has been proven as accurate and sensitive for detection of CNAs in hematological malignancies, particularly appearing advantageous for analyzing large sample sets in a time efficient fashion [15, 16]. MLPA demonstrated comparable detection rates to routine interphase FISH for aberrations being typically present in leukemia. Since it can detect also rare chromosomal aberrations it was suggested being applied as an initial test if routine cytogenetics is not possible or informative [17]. The SALSA P327_A1 and P327_B1 probe sets were designed to provide information on the copy number status of 24 and 29 genes located on chromosome 21, respectively. Six of the 31 probes targeting chromosome 21 are specific for *RUNX1* gene being located within the CRA. Other probes anneal to regions flanking the *RUNX1* gene described being duplicated or amplified in most cases with an iAMP21. In our hands low MLPA mean peak ratios within the CRA (<1.8) ruled the presence of an iAMP21 out, in all of these cases less than three additional *RUNX1* signals have been detected. By contrast, high MLPA mean peak (≥1.8) ratios should be taken as evidence for the presence of an iAMP21, even though encountering difficulties to detect this chromosomal abnormality by FISH performed from interphase preparations. Nevertheless, we cannot rule out that MLPA in some cases fails to accurately distinguish between BCP-ALL with 4 copies of a chromosome 21 and an iAMP21 due to certain variability between blast counts of different patient samples. On the other hand interphase FISH could not detect additional *RUNX1* signals in a total of six cases, shown to have gain of genetic material by MLPA. Even though we are aware of such methodological pitfalls, these cases unlikely harbored an iAMP21 since peak ratios obtained from both data sets were low to intermediate. Further investigation of such cases by whole genome techniques such as array based comparative genomic hybridization or, alternatively exome sequencing capable of assessing gene copy numbers would be desirable.

Depending on the chromosomal location of genes, MLPA peak ratios appeared heterogeneous in BCP-ALL with an iAMP21. The highest MLPA peak ratios and lowest variations between different patients were encountered within and close to the CRA while peak ratios often dropped towards telomeric regions. These observations are in line with the view that chromosome 21 aberrations in BCP-ALL with an iAMP21 are highly complex, exhibiting a stepwise increase in copy numbers towards the CRA and decrease of copy number towards the CRD, mapping centromeric and telomeric parts of chromosome 21, respectively [10].

Due to the lack of studies identifying individual genes, playing an important role in leukemogenesis or providing evidence that de-regulation of certain candidates would explain the poor outcome of patients with an iAMP21, the effect of gene load on mRNA expression within the CRA was also evaluated. In contrast to our expectations, this analysis revealed that patients with lowest gene dosages of *RUNX1*, *DYRK1A*, *ETS2* and *ERG* genes displayed the highest expression of the respective mRNA. These results need to be consolidated analyzing more patients. As part of a study to describe genomic alterations and gene expression in BCP-ALL with an iAMP21, Strefford *et al.* 2006 evaluated the expression of genes located in the CRA of eight cases and compared data with that of other BCP-ALL subtypes. Even though expression levels of the genes contained within the CRA were higher in BCP-ALL with an iAMP21 for almost half of the probe sets from the CRA no over-expression was observed, including the *RUNX1* gene. The authors concluded that these results could be explained by regulatory mechanisms such as epigenetics and biofeedback in BCP-ALL [9]. Strikingly, hyperdiploid leukemias, by definition harboring less gene copies on chromosome 21 than cases with an iAMP21, showed higher mRNA expression for the genes analyzed.

## Conclusions

Altogether, we confirmed that aberrations on chromosome 21 in BCP-ALL with an iAMP21 represent a high level of complexity but as a group exhibited similar MLPA peak ratio profiles and, furthermore provided evidence for the existence of gene regulatory mechanisms that might modulate the expression of amplified genes. MLPA profiles of CRA genes might be taken into account to facilitate diagnosis whenever metaphase nuclei for FISH are not available. However, for identification of an iAMP21 solely by MLPA next generation probe sets would need to include control fragments binding genes on chromosomes usually over-represented in hyperdiploid ALL.

## Methods

### Patients

A series of consecutive diagnostic samples from Brazilian childhood BCP-ALL patients collected between the years 2002 to 2012 were selected according to availability of material of good quality providing that bone marrow aspirates harbored at least 30 % of blast cells. The initial dataset contained samples from 606 patients. Out of those, 368 samples were retained for this study, excluding 238 cases based on the available results of mutually exclusive gene fusions (*ETV6/RUNX1*, *TCF3/PBX1*, *MLL-r* and *BCR-ABL1*). In addition five patients with ALL and Down syndrome were included.

As criteria for assigning as standard-risk or high-risk patients, age at diagnosis and WBC according to the Cancer Therapy Evaluation Program of the NCI were considered. Data collection and laboratory procedures have been evaluated and approved by the Research Ethics Committee from the National Cancer Institute (INCA) in Rio de Janeiro (CEP #005/06 and CAAE 626.268 at April 28, 2014). Informed consent was obtained from all subjects or their parents in accordance with the Declaration of Helsinki.

### Multiplex ligation-dependent probe amplification

The presence of an iAMP21 has been evaluated applying the MLPA SALSA P327_A1 (lot A1-0101) and P327_B1 (lot B1-0613) probe sets (MRC Holland, Amsterdam, The Netherlands). Tests fragments have been designed to evaluate the copy number status of genes distributed from centromeric to telomeric regions (q11.2-q22.3, 14.668-46.356) of chromosome 21. The P327_B1 probe set represents a modified version of the P327_A1 probe set in which 12 new probes for the *ERG* gene and 3 additional probes for chromosome 21 genes have been added. Four *RUNX1* specific probes (exon 10; 7; 6 and 4b) have been replaced while two probes (exon 9 and 2) remained unchanged. The size and location of most other test probes as well as reference probes has been redefined. Detailed information about P327_A1 and P327_B1 MLPA probe sets was downloaded from the MRC Holland website (http://www.mlpa.com); see Additional files 2 and 3, respectively. MLPA and capillary electrophoresis based amplification product separation (ABI 3130xl, Life Technologies, Carlsbad, CA) was performed according to the manufacturer’s instructions. Relative copy numbers were obtained after normalization of peaks against cord blood derived controls. Sequences were analyzed by the Gene Marker 2.2.0 software (Soft Genetics LLC, State College, PA).

### Fluorescence *in situ* Hybridization (FISH)

FISH was performed using the “LPH012 *TEL/AML1* Translocation, Dual Fusion Probe”, designed to detect the *TEL/AML1* fusion gene. Cells were processed according to the protocols instructions (Cytocell, Cambridge, UK). Cases were considered positive for an iAMP21 when ≥5 *RUNX1* signals were observed in at least 7 % of metaphase nuclei (standard deviation +/− 2 %). If metaphase FISH was not possible, an iAMP21 was identified as multiple copies of *RUNX1* in most interphase nuclei [1, 13].

### Reverse trancriptase quantitative (RT-q)PCR

Total RNA was isolated by Trizol reagent (Life Technologies, Carlsbad, CA) and used for cDNA synthesis by Super Script II Reverse Transcriptase (Life Technologies, Carlsbad, CA). RUNX1, ETS2, DYRK1A, ERG and, GAPDH transcripts used as an endogenous control were quantified by SYBR-green RT–qPCR (Bioline, London, UK) using the *Applied Biosystems 7300 Real-Time PCR System* (Applied Biosystems, Foster City, CA). For data depiction dCT values (CT target – CT reference) of biological replicates were calculated. The following primer combinations were used: *RUNX1*, forward 5'-GAGCTGAGAAATGCTACCGC-3', reverse 5'-GGTCAGAGTGAAGCTTTTCCC-3'; *ETS2*, forward 5'-TTCCAAAGAACCCCTGGCTG-3', reverse 5'-CGAACCTCTGCAGATTCACG-3'; *DYRK1A*, forward 5'- TGTAACCCCAAACGCAGTG-3', reverse 5'- ACCGATAAAAGCGACTCTGAA-3'; *ERG*, forward 5'-AAGTAGCCGCCTTGCAAAT-3', reverse 5'-GTGCCTTCCCAGGTGATG-3'; and *GAPDH*, forward 5'-TGACCCCTTCATTGACCTCA-3', reverse 5'-AGTCCTTCCACGATACCAAA-3'.

### Statistical analysis

Statistical analyses were performed using the Statistical Package for Social Sciences (SPSS), 18 (Chicago, IL), Graph Pad Prism 4.02 (La Jolla, Ca) and R (Foundation for Statistical Computing, Auckland, New Zealand) software packages. Confidence intervals of 95 % were used; *P*-values <0.05 were considered statistically significant. Chi-square and Fisher’s exact tests were used for comparative variable analyzes of demographic and clinical variables. Correlation and the strength of agreement between MLPA data were assessed by Pearson correlation coefficients and Kappa tests, respectively. Hierarchical clustering and principal component analyses (PCA) were created in R. For creation of heat maps MLPA values were scaled and centered using the scale function followed by clustering and plotting of normalized values using heat map .2 with default values. Patient groups from heatmaps were compared by unpaired, two sided Student’s T-tests. PCA was performed on scaled values using the prcomp() function with default parameters.

## Additional files

Additional file 1:
**Mean MLPA peak ratios in BCP-ALL obtained from test fragments of the MLPA SALSA P327-B1 probe set.**
Additional file 2:
**The size and location of each test and reference fragment of the MLPA SALSA P327_A1 probe set.**
Additional file 3:
**The size and location of each test and reference fragment of the MLPA SALSA P327_B1 probe set.**

## Abbreviations

BCP-ALL: B-cell precursor acute lymphoblastic leukemia
CNAs: Copy number alterations
CRA: Common region of amplification
CRD: Common region of deletion
dCT: Delta threshold cycle
DS: Down syndrome
FISH: Fluorescence *in situ* hybridization
Hyp + 21: Hyperdiploid ALL with extra copies of chromosomes 21
iAMP21: Intrachromosomal amplification of chromosome 21
P: Patient
PCA: Principal component analysis
PH: Patients with Hyp + 21
PT: Patients with DS
WBC: White blood cell count
